# The Cell Biology of the *Trichosporon*-Host Interaction

**DOI:** 10.3389/fcimb.2017.00118

**Published:** 2017-04-07

**Authors:** Cláudio Duarte-Oliveira, Fernando Rodrigues, Samuel M. Gonçalves, Gustavo H. Goldman, Agostinho Carvalho, Cristina Cunha

**Affiliations:** ^1^Life and Health Sciences Research Institute (ICVS), School of Medicine, University of MinhoBraga, Portugal; ^2^ICVS/3B's - PT Government Associate LaboratoryBraga/Guimarães, Portugal; ^3^Faculdade de Ciências Farmacêuticas de Ribeirão Preto, Universidade de São PauloSão Paulo, Brazil

**Keywords:** *Trichosporon*, trichosporonosis, virulence factors, host-pathogen interaction, antifungal immunity

## Abstract

Fungi of the genus *Trichosporon* are increasingly recognized as causative agents of superficial and invasive fungal disease in humans. Although most species are considered commensals of the human skin and gastrointestinal tract, these basidiomycetes are an increasing cause of fungal disease among immunocompromised hosts, such as hematological patients and solid organ transplant recipients. The initiation of commensal or pathogenic programs by *Trichosporon* spp. involves the adaptation to the host microenvironment and its immune system. However, the exact virulence factors activated upon the transition to a pathogenic lifestyle, including the intricate biology of the cell wall, and how these interact with and subvert the host immune responses remain largely unknown. Here, we revisit our current understanding of the virulence attributes of *Trichosporon* spp., particularly *T. asahii*, and their interaction with the host immune system, and accommodate this knowledge within novel perspectives on fungal diagnostics and therapeutics.

## Introduction

The *Trichosporon* genus comprises several basidiomycetous fungi that are ubiquitously found in nature (Colombo et al., 2011; Marine et al., 2015). Remarkably, their biology has evolved in a way to withstand different types of relationships with the human host. Most species are classical opportunistic pathogens, residing harmlessly as commensals on the skin and the gastrointestinal tract of healthy individuals (Zhang et al., 2011a; Gouba et al., 2014), where they are kept under surveillance by the immune system and through interactions with the resident microbiome. Alterations in the surrounding microenvironment may activate their pathogenic potential, leading to the subversion of immune tolerance and consequent tissue damage and overt disease. In this review, we discuss the latest advances to our understanding of the interplay between *Trichosporon* spp. and the human host, and that underpin their ecology as fungi exploring the interface between commensalism and pathogenesis.

## *Trichosporon* spp. and trichosporonosis

Among the *Trichosporon* species with pathogenic potential, *Trichosporon asahii, T. asteroides* and *T. mucoides* are responsible for the majority of cases of trichosporonosis, and represent important opportunistic pathogens among hematological patients (Colombo et al., 2011). Indeed, invasive trichosporonosis (IT) has historically been reported to present commonly as a disseminated infection in neutropenic hosts (Walsh et al., 1992, 1993). More recently, IT was mostly expressed as catheter-associated fungemias (Kontoyiannis et al., 2004), supporting their underappreciated role as cause of biofilm-related infections, also in other materials such as breast implants (Reddy et al., 2002). Of note, the use of high dose of corticosteroids was identified as one major predictor of mortality following IT (Kontoyiannis et al., 2004). IT may also occur in other contexts of immunosuppression (e.g., solid organ transplantation and autoimmune disease), newborns, and various conditions of debilitating disease (e.g., surgery, trauma and extensive burns) (de Almeida Junior and Hennequin, 2016). A recent epidemiological analysis of cancer patients has reported an increase in the incidence of invasive infections caused by *Trichosporon* spp. from 1.8 to 2.35 cases per 100,000 patient days (Chitasombat et al., 2012). This scenario may reflect the antifungal stewardship for neutropenic patients based on echinocandins, to which *Trichosporon* spp. are intrinsically resistant (Suzuki et al., 2010). Instead, and although no studies have compared different antifungal regimens in the management of IT, azole formulations may be superior to echinocandin- or amphotericin-based therapies (de Almeida Junior and Hennequin, 2016), despite the generally higher resistance displayed by non-*T. asahii* isolates (Rodriguez-Tudela et al., 2005). Specifically, the use of voriconazole significantly improved the prognosis of hematological patients, and is therefore the currently recommended first line of therapy (Arendrup et al., 2014).

The diagnosis of IT relies on the isolation and culture of the fungus from a clinical specimen. Even so, the direct examination of the clinical specimens often fails to identify the isolates at the species level. Direct sequencing of the intergenic spacer 1 (IGS1) region of the ribosomal DNA is the reference method for *Trichosporon* species identification (Sugita et al., 2002) and, more recently, matrix-assisted laser desorption/ionization (MALDI)-time-of-flight (TOF) mass spectrometry was also proposed as a valuable alternative for routine identification (de Almeida Junior et al., 2014). Although the performance of serum markers has not been thoroughly evaluated, patients with IT display significant levels of circulating β-D-glucan (Suzuki et al., 2010). Of note, and although *Trichosporon* spp. share antigenic properties with *Cryptococcus neoformans* (discussed in detail below), cross-reactions with the cryptococcal glucuronoxylomannan (GXM) antigen assay failed to yield a reasonable sensitivity (Lyman et al., 1995).

In addition to the life-threatening forms of trichosporonosis, the expansion of *T. asahii* colonizing the skin was reported in patients with atopic dermatitis (Zhang et al., 2011b), besides other superficial infections such as “white piedra,” which consists in the formation of hard nodules on hair shafts (Schwartz, 2004). Under certain geographic and climatic conditions, the repeated inhalation of arthroconidia from *T. asahii* can also cause summer-type hypersensitivity pneumonitis (SHP) (Sugita et al., 2004). In SHP, the repeated exposure to airborne fungal antigens induces lung inflammation characterized by alveolitis and non-necrotizing granulomas. *T. inkin, T. cutaneum, T. ovoides*, and *T. loubieri* are also considered important agents in superficial trichosporonosis (Marine et al., 2015).

## From a commensal to a pathogenic lifestyle

*T. asahii* is a morphologically and physiologically complex and adaptable yeast-like fungus, sharing several resemblances with *Candida albicans* (da Silva Dantas et al., 2016). Similar to other dimorphic fungi, and besides growing as a budding yeast, *T. asahii* is capable of filamentous growth, forming septate hyphae with abundant arthroconidia and blastoconidia (Marine et al., 2015). It can thrive in different host niches, including the skin, gut and oral mucosa, without causing disease. This ability is thought to rely on the acquisition of a developmental and phenotypic program toward a commensal state, in which specific virulence factors and the propensity for tissue invasion are suppressed (Pande et al., 2013). The mutualistic relationship of *T. asahii* with the human host also depends on its metabolic flexibility (Colombo et al., 2011). Indeed, this fungus is specialized in assimilating a large variety of carbon and nitrogen sources, a finding highlighting its close rapport with the local microbiome via nutrient recycling. The rewiring of metabolic pathways conveying a more flexible carbon assimilation strategy has been associated with the shift between the commensal and pathogenic states of *C. albicans* (Sandai et al., 2012; Childers et al., 2016). Furthermore, *C. albicans* has evolved mechanisms to bypass the nutritional limitations imposed by the host through the expression of micronutrient transporters (Crawford and Wilson, 2015) or redundant enzymes that use alternative micronutrients (Li et al., 2015). Thus, it is possible that, similarly to the basidiomycete *C. neoformans* (Kronstad et al., 2012; Choi et al., 2015), *T. asahii* also displays analogous metabolic traits in its pathogenic repertoire.

Changes in the availability of nutrients may influence the abundance and diversity of *Trichosporon* spp. and underlie significant dysbiosis of the gut mycobiota potentially leading to inflammatory pathologies, such as inflammatory bowel disease (Sokol et al., 2016). Supporting this concept, the recognition of fungal β-glucan by the innate immune receptor dectin-1 was found to be critical for the maintenance of the homeostatic interaction between the gut mycobiota and the host immune system (Iliev et al., 2012). In the absence of dectin-1-mediated signals, a dysbiosis toward the expansion of *Candida, Trichosporon* and *Saccharomyces* species in the gut resulted in increased susceptibility to severe ulcerative colitis. Likewise, the genetic deficiency of caspase recruitment domain family member 9 (CARD9), a signaling adapter downstream of dectin-1, rendered the gut microbiota unable to metabolize tryptophan into aryl hydrocarbon receptor ligands controlling the secretion of interleukin (IL)-22 (Lamas et al., 2016), a pivotal cytokine restraining pathogenic colonization by *C. albicans* (Zelante et al., 2013). Whether *T. asahii* colonization of the gut is likewise controlled by bacterial-derived tryptophan (or other) metabolites remains to be explored.

Collectively, the commensalism program displayed by *T. asahii* in the healthy gut is driven by both intrinsic (transcriptional landscape, cell morphology, metabolic plasticity) and extrinsic factors (competitive microbiome, diet, host immune status, and genetic profile). Although there is much to be learned about the factors affecting colonization at other sites, including the skin, it is likely that a plethora of similar factors also sustain the commensal state in these niches, and that the disruption of this metabolic homeostasis may instead promote virulence and contribute to disease.

## Virulence traits and evolution of phenotypic plasticity

**Figure 1:**
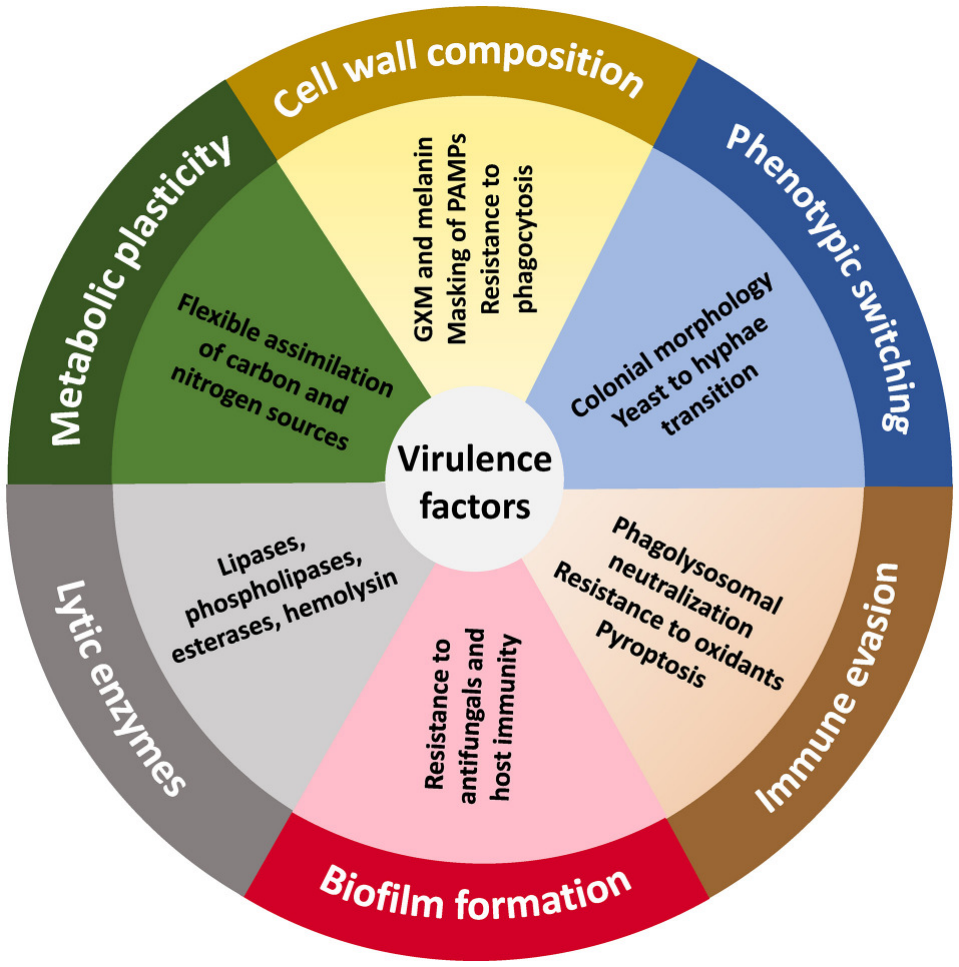
**The virulence landscape of ***Trichosporon*** species**. The ability to evade the host immune system and trigger disease is a polygenic trait in *Trichosporon* spp., particularly *T. asahii*, and that involves a dynamic regulation of biochemical, physiological, genetic and morphogenetic characteristics. GXM, glucuronoxylomannan.

The ability of *T. asahii* to invade the skin and other tissues requires several virulence traits, which include the yeast-to-hyphae transition, biofilm formation, the activity of lipases and proteases, and the dynamic composition of the cell wall (Figure 1). In this regard, recent polyploidization events within *Trichosporon* species are one important genomic feature underlying their phenotypic plasticity (Sriswasdi et al., 2016). These evolutionary traits enable *T. asahii* to adapt and survive in different host niches and support its successful transition to a pathogenic state. Both *Trichosporon* spp. and *Malassezia* spp. are examples of host-adapted commensals that possess, to some extent, the ability to activate these virulence features (Saunders et al., 2012). The acquisition of a pathogenic program by fungi is typically related with their ability to undergo phenotypic switching, a fast and reversible change of colonial morphology and/or microscopic features, which occurs in response to different environmental stimuli or stresses (Alby and Bennett, 2009). Accordingly, macro- and microscopic morphological differences were described for *T. asahii*, with the presence of rough, irregular colonies accompanied microscopically by a greater proportion of hyphae, and powdery colonies displaying a greater amount of conidia (Karashima et al., 2002).

The ability of *T. asahii* to form biofilms, structured microbial communities embedded in an extracellular polymeric substance, on synthetic surfaces such as central venous catheters, cardiac grafts and breast implants is critical for the establishment of IT (Bentubo and Gompertz, 2014). Although azoles have been reported to be effective against planktonic cells, they have failed to eradicate the preformed biofilms of *T. asahii* (Di Bonaventura et al., 2006). Besides influencing the response to antifungal therapy, these complex structures may also physically restrain the cellular immune system, thereby contributing to the establishment of infection. Although the pathogenic basidiomycete *C. neoformans* is not recognized as an efficient former of biofilms, it nevertheless possesses a vast infection toolkit that allows it to avoid the immune system and establish infection (Taylor-Smith and May, 2016). One of the most well-known virulence factors in this fungus is the production of the capsular polysaccharide GXM (Zaragoza et al., 2009). By protecting cells from phagocytosis and providing resistance to intracellular oxidative killing mechanisms, GXM collaborates in the evasion from the immune system (Taylor-Smith and May, 2016). Remarkably, and despite the lower extent, *Trichosporon* species are also able to produce GXM (Fonseca et al., 2009). However, although these share antigenic reactivity and contribute to resistance to phagocytosis, trichosporonal GXM displays smaller diameter and negative charge than that from *C. neoformans* that may directly affect polysaccharide assembly at the fungal surface. In support of a role for this polysaccharide in their pathogenic lifestyle, *T. asahii* isolates undergo morphological transition with a concomitant increase in the amount of secreted GXM after passage in a mouse model (Karashima et al., 2002). Accordingly, trichosporonal GXM was found to protect acapsular *C. neoformans* by restraining phagocytosis compared with yeast without detectable surface GXM (Fonseca et al., 2009), a finding suggesting GXM as a promising therapeutic target for the prevention of trichosporonosis (Hole and Wormley, 2012).

Melanin is another fungal cell wall component that significantly enhances the virulence of many important human pathogenic fungi (Nosanchuk et al., 2015). Regardless of the fungal species-specific structural characteristics of this pigment, melanization has been demonstrated as a crucial virulence factor during the interaction with the host immune system (Akoumianaki et al., 2016), to confer protection against antifungals (Fernandes et al., 2015) and to regulate capsule formation (Gish et al., 2016). Accordingly, the production of eumelanin from the phenolic precursor L-dihydroxyphenylalanine was described in *T. asahii* (Figueiredo-Carvalho et al., 2014). The expression of melanin in the fungal cell wall may be crucial in subverting the host immune system and avoiding intracellular clearance mechanisms such as the non-canonical autophagy pathway LC3-associated phagocytosis, which is critically required for fungal killing (Akoumianaki et al., 2016). Taken as a whole, changes in the polysaccharide composition of the cell wall may modify the sensitivity of *T. asahii* to environmental stresses and antifungals (Ene et al., 2012a,b). More important, these changes may alter the expression and presentation of critical pathogen-associated molecular patterns (PAMPs) readily recognized by the innate immune system, with profound consequences to the interaction with the host. Indeed, the fungal cell wall of *T. asahii* contains galactosaminogalactan (GAG) (Lee et al., 2016), an immunosuppressive polysaccharide able to inhibit neutrophil infiltration and promote fungal development (Fontaine et al., 2011), in part by masking β-glucans from recognition by dectin-1 (Gravelat et al., 2013). It remains to be explored whether trichosporonal GAG also functions by inducing the immunoregulatory cytokine IL-1 receptor antagonist (IL-1Ra) (Gresnigt et al., 2014), ultimately pointing to the possibility of targeting IL-1Ra in trichosporonosis.

The study of extracellular export of specific enzymes has contributed to the understanding of ecological characteristics of pathogenic yeasts (Gacser et al., 2007). Hemolysins, proteases, and lipases allow destabilization of host membranes and degradation of host connective tissues, cleavage of host immunity-associated proteins, ultimately aiding in the acquisition of nutrients and the escape from the host immune system. Likewise, a considerable predominance of lipases and phospholipases was detected in the extracellular enzyme profiles of different species of *Trichosporon* (Bentubo and Gompertz, 2014). This may explain the temperature kinetics of proteases and phospholipases, which are favored at 37°C and may reflect their prominent role during fungal pathogenesis. Of note, clinical isolates of *T. asahii* were also found to display hemolysin activity (Sun et al., 2012), a trait that is in line with the recent identification of candidalysin, a secreted protein with host cell lytic activity secreted by *C. albicans* (Moyes et al., 2016). During active penetration of tissues, expression of superoxide dismutases may also be relevant for the detoxification of reactive oxygen species released during tissue damage (Zhang et al., 2016).

## The *Trichosporon*-host interaction

**Figure 2:**
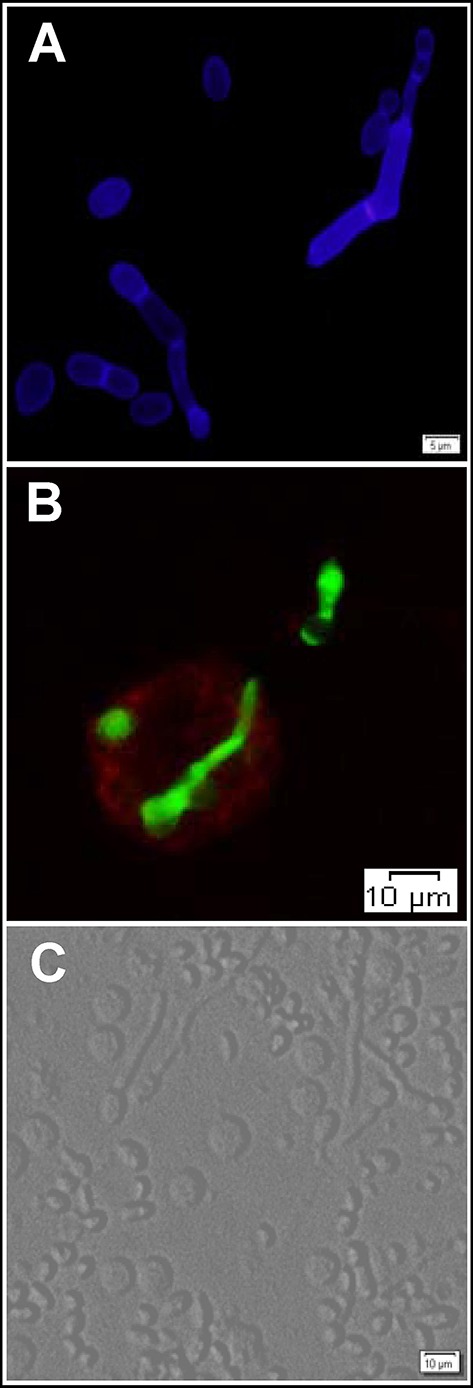
**The interaction of ***T. asahii*** with host immune cells. (A)** Blastoconidia and pseudohyphae morphotypes of *T. asahii* stained with Calcofluor White. **(B)** Phagocytosis of *T. asahii* by human macrophages. Fungal structures and macrophages were stained with fluorescein isothiocyanate (FITC) and phycoerythrin (PE)-conjugated anti-CD14 antibody, respectively. *Trichosporon* cells were incubated overnight at 4°C with FITC (Sigma-Aldrich) at a concentration of 0.1 mg/mL in 0.1M Na_2_CO_3_ buffer. **(C)** Bright-field microscopy visualization of human peripheral blood mononuclear cells aggregated around the different morphotypes of *T. asahii*.

The immune recognition of fungi, including *Trichosporon* spp., is mediated by cells of the innate immune system, including monocytes/macrophages, dendritic cells, and polymorphonuclear neutrophils (Figure 2). These cells express a vast repertoire of pattern recognition receptors (PRRs) in different subcellular compartments, including Toll-like receptors (TLRs), C-type lectin receptors and nuclear oligomerization domain-like receptors. These receptors sense molecular motifs and drive the secretion of proinflammatory mediators that polarize adaptive immune responses. The efficiency of fungal sensing also relies on the action of collectins, ficolins, pentraxins, and complement components that act as opsonins and facilitate the interaction of phagocytes with fungi.

The cell wall is the main source of PAMPs, including mannans, β-glucans and chitin, owing to its dynamic composition and structural properties according to morphotype, growth stage, and environmental conditions (Erwig and Gow, 2016). Because of its crucial role in recognizing cryptococcal GXM and restricting fungal growth (Yauch et al., 2004), TLR2 may be one major innate immune receptor involved in the recognition of *Trichosporon* species. Although cryptococcal GXM was shown to markedly inhibit proinflammatory responses (Piccioni et al., 2013), whether trichosporonal GXM shares these traits is unknown. Because mutations in myeloid differentiation primary response gene 88 (MyD88) do not lead to fungal disease in humans (Picard et al., 2011), receptors other than TLRs and the IL-1 receptor family may also be involved in the recognition of *Trichosporon* species. Indeed, dectin-1, rather than dectin-2, was reported as a crucial receptor for *T. asahii* (Higashino-Kameda et al., 2016). By controlling the expansion of T helper (Th)17 cells and downstream recruitment of neutrophils, dectin-1 engagement by *T. asahii* was found to play a significant role in the pathogenesis of SHP. The absence of documented cases of IT in humans carrying CARD9 mutations (Lanternier et al., 2015; Rieber et al., 2016) may suggest a particular involvement of dectin-1 in *Trichosporon*-induced allergy. On the other hand, galectin-9, a protein with binding specificity toward β-galactoside sugars, was shown to suppress *T. asahii*-induced lung inflammation by expanding immunosuppressive inflammatory monocytes, ultimately ameliorating Th1/Th17 cell-mediated hypersensitivity pneumonitis (Arikawa et al., 2010).

Besides membrane-bound PRRs, human macrophages infected with *T. asahii* were found to display a transcriptional profile enriched for pentraxin 3 (PTX3) (Cong et al., 2015). PTX3 is a fluid-phase PRR that displays ancestral antibody-like properties, recognizing and interacting with self and nonself molecular patterns, and displaying opsonic and antifungal activities at the crossroad between complement and Fcγ receptor (FcγR)-mediated recognition (Moalli et al., 2010). PTX3 has been shown to bind to several fungal pathogens, including *A. fumigatus* and *Paracoccidioides brasiliensis* (Garlanda et al., 2016), although its ability to bind *Trichosporon* spp. has not been explored yet. However, given that cryptococcal GXM impairs immune function via the direct stimulation of the inhibitory FcγRIIB (Monari et al., 2006), and that genetic variants in FcγRIIIA increase the risk for human immunodeficiency virus (HIV)-associated cryptococcal disease (Rohatgi et al., 2013), it is plausible that a PTX3-FcγR axis is also in play during the immune response to trichosporonal GXM. This hypothesis is further supported by the finding that the binding and phagocytosis of *T. beigelii* by neutrophils was a complement-dependent process enhanced by granulocyte-macrophage colony stimulating factor (GM-CSF) (Richardson and Chung, 1997), with consequences on fungal killing (Lyman et al., 1994). M-CSF displayed comparable effects, stimulating the antifungal activity of monocytes against *T. asahii*, partly via the production of tumor necrosis factor (TNF)-α (Sasaki et al., 2000). Likewise, survival in a neutropenic mouse model of trichosporonosis was improved by GM-CSF treatment, a finding attributed to increased neutrophil counts and TNF-α production in the lungs (Muranaka et al., 1997).

Recently, it has become clear that the innate immune response not only confers immunity, but can be trained prior to exposure to deliver immunological memory (Quintin et al., 2012), a paradigm that was previously only attributed to the adaptive immune system. Although the underlying molecular and immunometabolic processes leading to “trained immunity” have been vastly dissected for β-glucan (Cheng et al., 2014; Saeed et al., 2014; Arts et al., 2016), whether this carbohydrate also confers memory to *Trichosporon* spp., or whether other cell wall components possess identical properties remains to be explored.

Upon the ingestion of the fungal cell, maturation of the phagosome into the phagolysosome is fundamental to clearance. The promotion of fungal killing relies on cationic peptides, hydrolytic enzymes, and reactive oxygen (ROS) and nitrogen species, which are accumulated in acidic vacuoles (Erwig and Gow, 2016). ROS are specifically required to control *Trichosporon* spp. by phagocytes since invasive disease has been documented in patients with chronic granulomatous disease (CGD) (Moylett et al., 2003; Wynne et al., 2004), and because defective myeloperoxidase-dependent oxidative systems were found to increase susceptibility to experimental *T. asahii* infection (Aratani et al., 2000).

Thus, to withstand these harsh environments, fungi have devised strategies to allow their survival by manipulating and escaping from phagocytes. These mechanisms generally rely on the inhibition of phagosome maturation, blocking of phagosomal acidification, or escape from the phagosome (da Silva Dantas et al., 2016). Although not much is known about the subverting mechanisms of *T. asahii*, based on the similarity of virulence traits, certain parallelisms with other fungi can be drawn. For example, dihydroxynaphthalene-melanin from *A. fumigatus* was reported to inhibit lysosomal fusion (Thywißen et al., 2011). Likewise, and because the deposition of eumelanin in the cell wall is essential for the pathogenicity of *Trichosporon* spp., it is plausible that a similar inhibitory mechanism takes place. In addition, *T. asahii* possesses a battery of scavenging enzymes, including catalase and superoxide dismutases (Zhang et al., 2016) that may allow its survival inside the phagosome. The escape from the innate immune system may also be conferred by the induction of pyroptosis—a form of programmed cell death that is dependent on caspase-1 activation of the inflammasome in host phagocytes (Krysan et al., 2014). In support of this, *T. asahii* was found to be a potent inducer of the proinflammatory cytokine IL-1β, whose production also relies on its proteolytical cleavage by caspase-1 (Cong et al., 2015). Taken together, these observations highlight the well-adapted cell biology of *T. asahii* to resist the antifungal effector mechanisms of phagocytes, which inevitably may also condition the activation of adaptive immunity modules.

## Conclusions and future perspectives

Recent studies have attempted to characterize the dynamic interaction of *Trichosporon* spp. with the host immune system. These efforts have shed light on the diversity and sophistication of the features through which *Trichosporon* spp. regulate the immune response and either establish a commensal program or trigger overt disease. While the virulence landscape of many fungi is generally insufficient to overcome a competent immune system, they are likely pivotal in driving pathogenesis in susceptible hosts. Therefore, a better understanding of the virulence traits activated during acquisition of a pathogenic transcriptional program is certain to provide exciting insights that will allow to pin down crucial functional networks defining the outcome of the host-fungus interaction. Specifically, the structural characterization of the cell wall and surface, and the precise definition of its immunological properties is expected to be a cutting-edge research topic in this area. In addition, understanding the mechanisms by which *Trichosporon* spp. evade the host antimicrobial defenses could lead to the identification of new therapeutic (and immunotherapeutic) targets and the development of safe vaccines. Since the availability of antifungal agents is still limited and no vaccine is currently available, this goal is of great importance for the treatment of fungal infections, since conventional antifungal therapy is expected to benefit from the association with adjuvant immunotherapy.

## Author contributions

All named authors have made an active contribution to the conception and design, and/or drafting of the paper, and have approved the final version submitted for publication.

### Conflict of interest statement

The authors declare that the research was conducted in the absence of any commercial or financial relationships that could be construed as a potential conflict of interest.
